# The Eco-Physiological Role of *Microcystis aeruginosa* in a Changing World

**DOI:** 10.3390/microorganisms10040685

**Published:** 2022-03-23

**Authors:** Leda Giannuzzi, Marcelo Hernando

**Affiliations:** 1 Area of Toxicology, School of Exact Sciences, National University of La Plata (UNLP), La Plata 1900, Argentina; leda@biol.unlp.edu.ar; 2 Department of Radiobiology, National Atomic Energy Commission, San Martin 1650, Argentina; 3 Red de Investigación de Estresores Marinos-costeros en América Latina y el Caribe (REMARCO), Mar del Plata 7602, Argentina

## 1. Introduction

Among the bloom-forming cyanobacteria, *Microcystis aeruginosa* is one of the most harmful species. The prevalence of toxic species and their toxicity profiles vary each year depending on environmental variables, despite their dynamics being not well understood. Imbalances in nutrient concentrations, the greater frequency and intensity of higher average temperatures and less severe winters, and the alternation between periods of positive and negative precipitation anomalies were identified as conditions favoring the prevalence of *M. aeruginosa*. Similarly, dry periods (corresponding to a prevailing La Niña condition) were even more conducive to the formation of harmful algal blooms of pronounced intensity. Moreover, toxin production is temperature-dependent and some strains are capable of producing multiple microsystins (MCs). Thus, the role of environmental variables in controlling the genetic expression of toxin synthetase of individual toxin-producing genes is still unknown.

## 2. Increased Temperature and Solar Ultraviolet Effects (UVR)

Great efforts have been put to assess the impact on diverse organisms of both UVR and global warming due to the input of greenhouse gas. Solar ultraviolet A (UVA) radiation (315–400 nm) has also been implicated in the inhibition of primary production and plays an additional role in activating UV-damage-repair mechanisms. UVR is currently considered a stressor for aquatic organisms even under its normal levels, and for cyanobacteria in particular. Very little is known about the interactive effects of UVR and temperature that may act synergic or antagonistically [1]. Higher temperature increases growth rate of cyanobacteria by accelerating metabolism, while at the ecosystem level it favors thermal stratification that promotes biomass accumulation at the water surface [2]. Global warming increases water temperatures and thus the stratification of water bodies depending on their morphometry and wind speed. By reducing the thickness of the upper mixed layer, organisms are exposed to a higher dose of UVR with physiological consequences like increased reactive oxygen/nitrogen species, lipid damage, and antioxidant protection [1].

### 2.1. Fatty Acids (FAs) Composition

The study by de la Rosa et al. [3] demonstrated differential sensitivity of omega 3 (ω3) fatty acids (FAs) in *M. aeruginosa* after two days of exposure to elevated temperature (29 °C) compared with the control (26 °C). Thus, although no significant differences were found in unsaturated/saturated FAs, differential sensitivity of ω3 versus ω6 was observed at 29 °C.

Previous studies have shown that UVR can affect FAs in cyanobacteria by damaging cell integrity, including oxidative damage to unsaturated or polyunsaturated lipids. According to [1], the lower lipid peroxidation of selected unsaturated fatty acids (UFAs) and the high capacity of scavenger systems could probably explain the high adaptability of *M. aeruginosa* to high UVR stress at high temperatures. It was evident that selected polyunsaturated fatty acids (PUFAs) (mainly ω6) play an important role in adaptation to high temperatures, in addition to the increased activity of enzymatic antioxidants that shifts temperature growth from 26 to 29 °C.

### 2.2. Microcystin

Dziallas and Grossart [4] hypothesized that MCs function as radical scavengers. Moreover, several studies showed increased sensitivity of MC-deficient mutants under high light and oxidative stress conditions, supporting the hypothesis of an antioxidant function of MC. Giannuzzi et al. [2] showed a decreased rate of several MCs (D-Leu1; Leu1, A sp3; Leu1, Glu(OCH3)6; M(O)1) after several days of exposure to high temperatures (29 °C).

Recently, Malanga et al. [5] showed effective antioxidant activity for [D-Leu1] MC-LR against water-soluble radicals in the first in vitro results related to the role of MC as an antioxidant. These results confirm the suggestions of the above authors regarding the decrease in the concentration of MC under environmental conditions requiring adaptation of *M. aeruginosa*. However, Giannuzzi et al. [2] also demonstrated a significant increase in one of the MCs produced by *M. aeruginosa*. This was the case for MC-LR, one of the most potent toxins in the world, when cultures were exposed to elevated temperature (29 °C). This suggests that the antioxidant function of the different MCs needs further investigation.

Regarding UVR exposure, Hernando et al. [6] also showed that short-term exposure (4 h) of *M. aeruginosa* to UVA resulted in a significant decrease in [Leu1] MC-LR (*p* < 0.05) when UVR + photosynthetic available radiation (PAR) doses reached 11,173 kJ m^−2^ (a UVBR dose of 74.4 kJ m^−2^).

## 3. Microcystin Brain Toxicity

MCs are potent hepatotoxins and tumor promoters that utilize an active carrier mechanism in the liver, considered one of the most dangerous toxins groups worldwide and being the leading cause of human fatalities. Despite the large amount of information available, interest in MCs continues to grow, as they are known to pose a threat to livestock, fisheries, aquaculture, human health, and wildlife through exposure via drinking, environmental, and recreational waters.

Although most studies to date have focused on the effects of MC on the liver, few studies have evaluated potential effects on the brain. A group of experts has called for studies on toxicity to neurodevelopment to be conducted for all major groups of cyanotoxins, as this topic remains largely unexplored

Moreover, in mammals, MCs exhibit potent neurotoxic activity that can cause significant behavioral changes, loss of neurons, and severe morphological changes, and on the other hand, they induce oxidative stress at the brain level and changes in the neuronal cytoskeleton. Effects such as memory loss after exposure to MC-LR are also highlighted [7]. In addition, ultrastructural brain changes have been observed in rats brains after exposure to MC-LR (10 μg/kg body weight/day) during gestation and lactation [8]. Histological and ultrastructural lesions and severe oxidative damage were also observed in the hippocampus treated with MC-LR (10 μg/L per 1μL) [9]. Recently, evaluating the chronic effects of MC in rats, it has been determined that MC-LR and D-Leu1 MC-LR do not reach the same brain structures, with the hippocampus being the area most affected by D-Leu1 MC-LR (Hernando, personal communication).

## 4. Microcystin Depuration Improves

Physicochemical control methods include UVR, ultrasound, and algaecides such as copper sulphate and chlorination. However, these measures are rarely applied on a large scale due to their high cost and potential secondary pollution. Therefore, the development of environmentally friendly and economical methods to control harmful algae blooms (HABs) is of both theoretical and practical importance. The release and degradation of MC-LR, produced by *M. aeruginosa* during the flocculation and lysis phases, have also been studied because of its importance in controlling and treating HABs without causing adverse effects.

Great efforts are currently being made to implement new, more advanced measures to eliminate cyanobacteria and toxins in a water treatment plant. Conventional treatment approaches such as coagulation/flocculation of cells by physical retention or chemical oxidation employing chemical compounds such as aluminum polychloride present various practical, economic or environmental disadvantages. A new emerging technologies, such as advanced oxidation processes (AOPs), new materials, such as activated carbon, chitosan and others, have great potential to resolve this issue. However, their efficiency, applicability, economic, practical and environmental aspects must be studied in depth. Finally, a combination of different treatment methods, including a multi-barrier approach, needs to be considered to produce drinking water.

## 5. Conclusions and Future Remarks

Considering the importance of water bodies where recurrent blooms of *M. aeruginosa* occur, it is essential to understand the relationship between environmental variables and the toxicity in them for the management and implementation of prevention measures related to the distribution and consumption of drinking water. Cyanobacteria were able to develop diverse and highly effective eco-physiological adaptations and strategies for ensuring survival and dominance in aquatic environments undergoing natural and human-induced environmental change. Thus, changes in metabolism and physiology may have serious consequences over the entire ecosystem. Considering that MCs are chemically very stable, and the rapid chemical modification of its structure or its antioxidant function against various environmental changes, these toxins can finally end up in human food through the food chain or drinking water, and the chronic impact of accumulated MCs on human health cannot be ignored; not only on the liver, but also in the brain, depending on the MC family as a function of the pre-exposure of *M. aeruginosa* to changing environmental variables. Other mechanisms of adaptation to changes in environmental variables may also be related to the composition of the cell lipid membrane. Shifts in FAs composition in a food web due to cyanobacterial blooms could potentially affect zooplankton/fish health by altering their metabolic processes, a pathway that is only recently being explored in trophic ecology.

This issue summarizes the current knowledge of *M. aeruginosa*, focusing on physiology, toxin production, toxic effects and depuration methods. It discusses how these factors influence the ecology of this globally important cyanobacterium, identifies a number of gaps in our knowledge, and provides a list of high-priority research topics. The aim of the Special Issue on *M. aeruginosa* is to establish how, and to what extent if any, changes in environmental conditions can influence the global dominance of cyanobacteria.

## Data Availability

We choose to exclude this statement due the study did not report any data.
